# Nogo-B receptor promotes the chemoresistance of human hepatocellular carcinoma via the ubiquitination of p53 protein

**DOI:** 10.18632/oncotarget.7091

**Published:** 2016-01-31

**Authors:** Chengyong Dong, Baofeng Zhao, Fei Long, Ying Liu, Zhenzhen Liu, Song Li, Xuejun Yang, Deguang Sun, Haibo Wang, Qinlong Liu, Rui Liang, Yan Li, Zhenming Gao, Shujuan Shao, Qing Robert Miao, Liming Wang

**Affiliations:** ^1^ Division of Hepatobiliary and Pancreatic Surgery, Department of General Surgery, The Second Affiiated Hospital of Dalian Medical University, Dalian, Liaoning, China; ^2^ Key Laboratory of Separation Science for Analytical Chemistry, National Chromatographic R & A Center, Dalian Institute of Chemical Physics, Chinese Academy of Sciences, Dalian, China; ^3^ Department of Oncology, The Affiliated Zhongshan Hospital of Dalian University, Dalian, China; ^4^ Institute of Cancer Stem Cell, Dalian Medical University, Dalian, China; ^5^ Key Laboratory of Proteomics, Dalian Medical University, Dalian, China; ^6^ Division of Pediatric Surgery, Department of Surgery, Children's Research Institute, Medical College of Wisconsin, Milwaukee, WI, USA; ^7^ Division of Pediatric Pathology, Department of Pathology, Children's Research Institute, Medical College of Wisconsin, Milwaukee, WI, USA; ^8^ Key Laboratory for Biomedical Effects of Nanomaterials and Nanosafety, National Center for Nanoscience and Technology of China and Institute of High Energy Physics, Chinese Academy of Sciences (CAS), Beijing, China

**Keywords:** NgBR, chemoresistance, HCC

## Abstract

Nogo-B receptor (NgBR), a type I single transmembrane domain receptor is the specific receptor for Nogo-B. Our previous work demonstrated that NgBR is highly expressed in breast cancer cells, where it promotes epithelial mesenchymal transition (EMT), an important step in metastasis. Here, we show that both *in vitro* and *in vivo* increased expression of NgBR contributes to the increased chemoresistance of Bel7402/5FU cells, a stable 5-FU (5-Fluorouracil) resistant cell line related Bel7402 cells. NgBR knockdown abrogates S-phase arrest in Bel7402/5FU cells, which correlates with a reduction in G1/S phase checkpoint proteins p53 and p21. In addition, NgBR suppresses p53 protein levels through activation of the PI3K/Akt/MDM2 pathway, which promotes p53 degradation via the ubiquitin proteasome pathway and thus increases the resistance of human hepatocellular cancer cells to 5-FU. Furthermore, we found that NgBR expression is associated with a poor prognosis of human hepatocellular carcinoma (HCC) patients. These results suggest that targeting NgBR in combination with chemotherapeutic drugs, such as 5-FU, could improve the efficacy of current anticancer treatments.

## INTRODUCTION

Hepatocellular carcinoma (HCC) is the second leading cause of cancer-related death and the fifth most common malignancy worldwide [1–3]. Curative interventions such as transplantation, resection, and thermal ablation are applicable for the 30% of patients whose tumors or liver function meet defined criteria [4]. Patients with advanced HCC or liver disease are not suitable for these curative interventions and require systemic chemotherapy [5]. However, these patients frequently develop drug resistance causing their treatments to fail [6, 7].

The mechanisms underlying cancer cell chemoresistance are complex and numerous [8–10], including increased drug efflux, reduction in drug absorption, changes of anticancer drugs’ targets, decreased drug activity, tumor hypoxia, enhancement of DNA repair following damage, abrogation of apoptotic effector pathways by altered gene expression, and changes in signaling pathways. One of the most important causes of resistance is increased expression of the drug transporter family known as the ATP-binding cassette (ABC) transporters. The most common member of this family is the ABCB1 (also known as MDR1 (multidrug resistance gene 1) which encodes for P-glycoprotein. Overexpression of P-glycoprotein is associated with lower accumulation of doxorubicin in HCC cells and with worse prognosis in patients [10]. EMT is also an important process by which HCC acquire 5-FU chemoresistance [11]. Furthermore, the activation of an Oct4-AKT-ABCG2 pathway has been identified to enhance drug resistance in HCC [12, 13]. A comprehensive understanding of mechanisms of chemoresistance in HCC could present opportunities to improve the response to cytotoxic agents and improve clinical outcomes.

The Nogo isoforms-A, -B and -C are members of the reticulon family of proteins. Nogo-A and Nogo-C are highly expressed in the central nervous system (CNS), while Nogo-B is found in most tissues [14, 15]. Nogo-B was previously identified as highly expressed in caveolin-1 enriched microdomains of endothelial cells (EC) [16]. The amino terminus (residues1–200) of Nogo-B (AmNogo-B) serves as a chemoattractant for EC [16]. NgBR was identified as a receptor specific for AmNogo-B by an expression cloning approach [15, 17]. We have previously demonstrated that NgBR is necessary for *in vivo* angiogenesis in zebrafish via the Akt pathway [18] and NgBR is highly expressed in human breast invasive ductal carcinoma [19]. NgBR breast tumor cell expression is highly correlated with expression of estrogen receptor and survivin [19]. Further study showed that NgBR promotes EMT in breast tumor cells, [20] but any role of NgBR in cancer drug resistance is still unclear. Here, we show that NgBR depletion sensitizes the 5-FU-resistant Bel7402/5FU cells to 5-FU treatment via disruption of the PI3K/Akt/MDM2 signaling pathway and stabilization of p53 protein. Our results suggest that NgBR is a potential novel drug target that can be used to increase the efficacy of conventional chemotherapeutic agents.

## RESULTS

### NgBR expression is increased in the drug resistant human HCC cells

**Figure 1:**
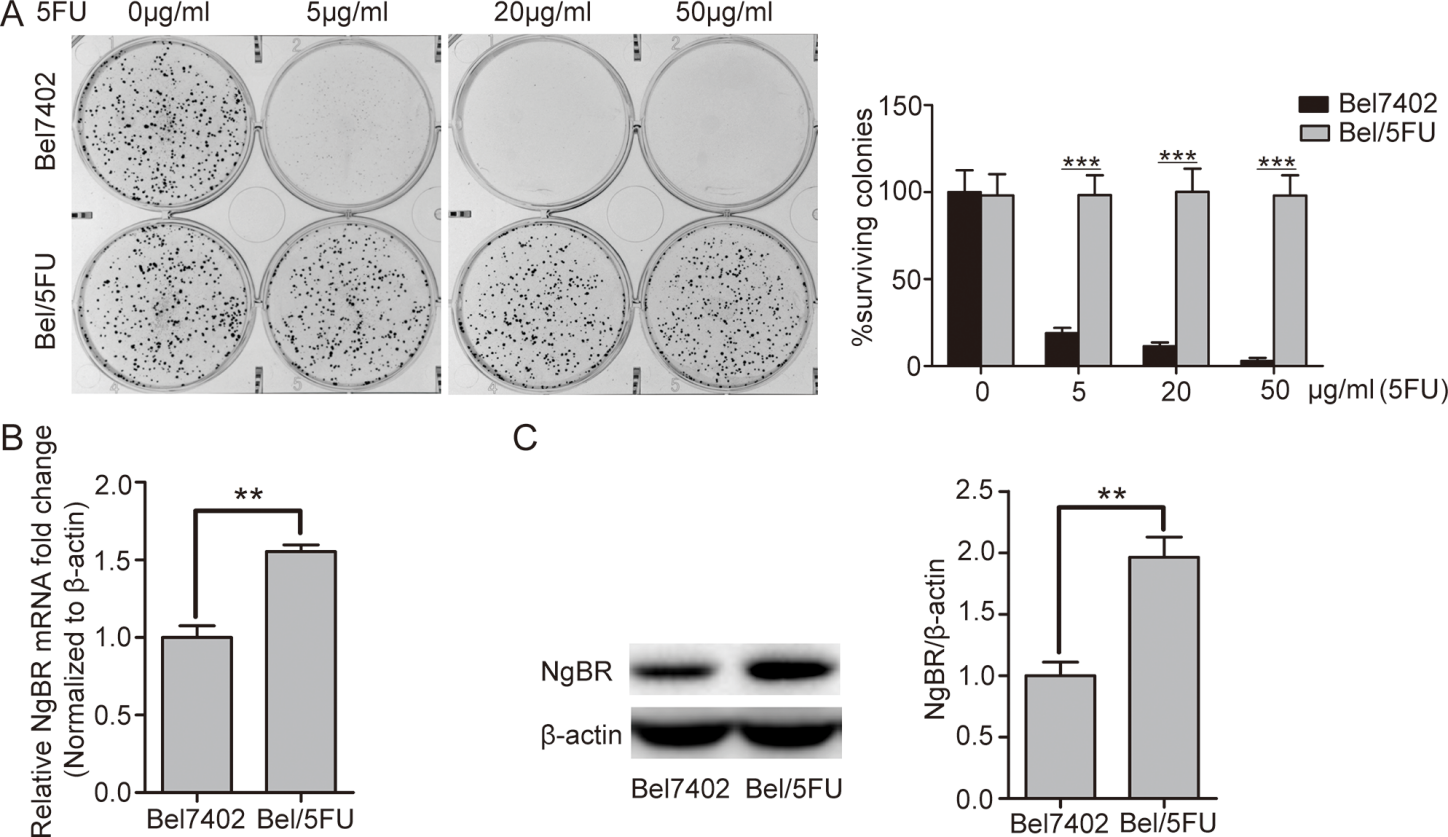
NgBR is highly expressed in the chemoresistant Bel/5FU cells (**A**) The 5-FU resistant phenotype was confirmed in Bel/5FU cells. Clonogenic survival assay was used for measuring clonogenicity of Bel7402 and Bel/5FU cells treated with different concentrations of 5-FU (0, 5, 20, and 50 μg/mL) (left panel). The number of untreated cells is set as 100%. The results were analyzed and show the average percentage of surviving colonies (right panel). (**B**) High mRNA level of NgBR was detected in chemoresistant Bel/FU cells. mRNA level of NgBR was analyzed using real-time RT-PCR and normalized to the β-actin. (**C**) High NgBR protein level was detected in chemoresistant Bel/5FU cells. Protein level was monitored using western blot (left panel). B and intensities were quantified using Image Lab 5.0 software and were normalized to β-actin (right panel). The data are presented as the mean ± SD of three independent experiments. (**P < 0.01, ***P < 0.001).

To confirm the 5-FU chemoresistance phenotype in Bel/5FU cells, the HCC parental cells (Bel7402) and the chemoresistant HCC cells (Bel/5FU) were treated with the indicated concentrations of 5-FU, and cell proliferation and survival were assessed using clonogenic survival assays. As shown in Figure 1A, compared with the Bel7402 control cells, the Bel/5FU cells were resistant to 5-FU. Then, the mRNA and protein levels of NgBR were evaluated in both cell lines. As shown in Figure 1B and 1C, both the NgBR mRNA and protein levels were increased in the Bel/5FU cell lines. These results indicate that higher NgBR expression is associated with chemoresistance in human HCC cell lines.

### NgBR knockdown decreases the chemoresistance of Bel/5FU cells *in vitro*

**Figure 2:**
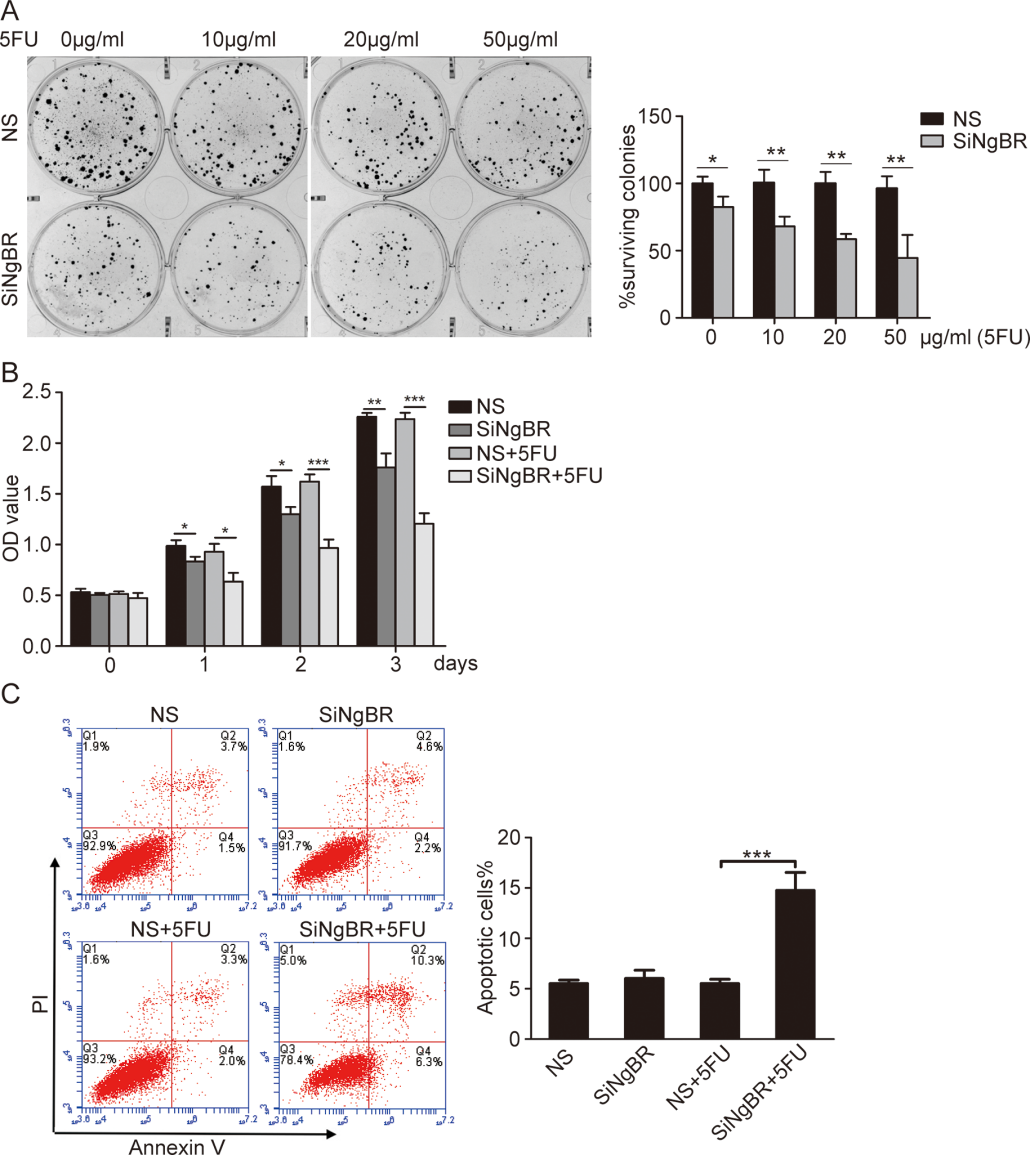
Knockdown of NgBR decreases the chemoresistance of Bel/5FU cells to 5-FU (**A**) Knockdown of NgBR decreases the clonogenenicity of Bel/5FU cells. Clonogenic formation assay was used for measuring clonogenicity of Bel/5FU cells treated with different concentrations of 5-FU (0, 10, 20, and 50 μg/mL) (left panel). The number of untreated cells is set as 100% and the results show the average percentage of surviving colonies (right panel). (**B**) Knockdown of NgBR decreases Bel/5FU cell viability. Cell viability was analyzed by CCK8 assay in Bel/5FU cells treated with 5-FU (0 and 50 μg/mL) for different times 24 h, 48 h, and 72 h. (**C**) Knockdown of NgBR increases apoptosis of Bel/5FU cells induced by 5-FU. The apoptotic cells were detected by Annexin V-PI dual staining. Representative data from three independent experiments are shown in left panel, the total number of cells in the Q2 and Q4 quadrant was regarded as apoptotic cells. Percentages of apoptotic cells are shown in the bar graph (right panel). The data are means ± SD of three independent assays. (*P < 0.05,**P < 0.01, ***P < 0.001).

To determine whether knockdown of NgBR could decrease the chemoresistance of Bel/5FU cells, NgBR siRNA was used to silence the expression of NgBR. The efficiency of transfection was evaluated by real-time RT-PCR and western blot. The results demonstrate that all three NgBR siRNAs (S1, S2, and S3) effectively reduce the mRNA and protein levels of NgBR(Supplementary Figure S1A and S1B). Clonogenic survival assay showed knockdown of NgBR by all three siRNAs (S1, S2, and S3) reduces colony formation of Bel/5FU cells in response to 5-FU treatment (Supplementary Figure S1C). We used S1 for the following NgBR siRNA knockdown experiment. Clonogenic assay further showed that knockdown of NgBR by siRNA reduces the colony number in response to 5-FU treatment in a dose dependent manner (Figure 2A). In addition, CCK-8 assay results also revealed knockdown of NgBR decreases the cell survival rate of Bel/5FU cells at all 5-FU treatment time points (Figure 2B). These results indicate that NgBR knockdown decreases the chemoresistance of Bel/5FU cells.

To confirm that apoptosis contributes to the inhibitory effects of NgBR knockdown on Bel/5FU cell chemoresistance, we used Annexin V-FITC/ propidium iodide (PI) staining-based fluorescence activated cell sorting (FACS) analysis to examine cell apoptosis. Knockdown of NgBR did not increase apoptosis of Bel/5FU cells, and 5-FU treatment alone did not increase apoptosis of the Bel/5FU control cell (non-specific siRNA or NS) either (Figure 2C). However, 5-FU increased apoptosis of Bel/5FU NgBR knockdown cells (siNgBR). AO/EB staining incorporation assay indicates that NgBR knockdown decreased the chemoresistance of the Bel/5FU cell by increasing 5-FU induced HCC cell apoptosis (Supplementary Figure S2).

### NgBR knockdown induces the abrogation of S-phase arrest of Bel/5FU cells by increasing p53 protein level

**Figure 3:**
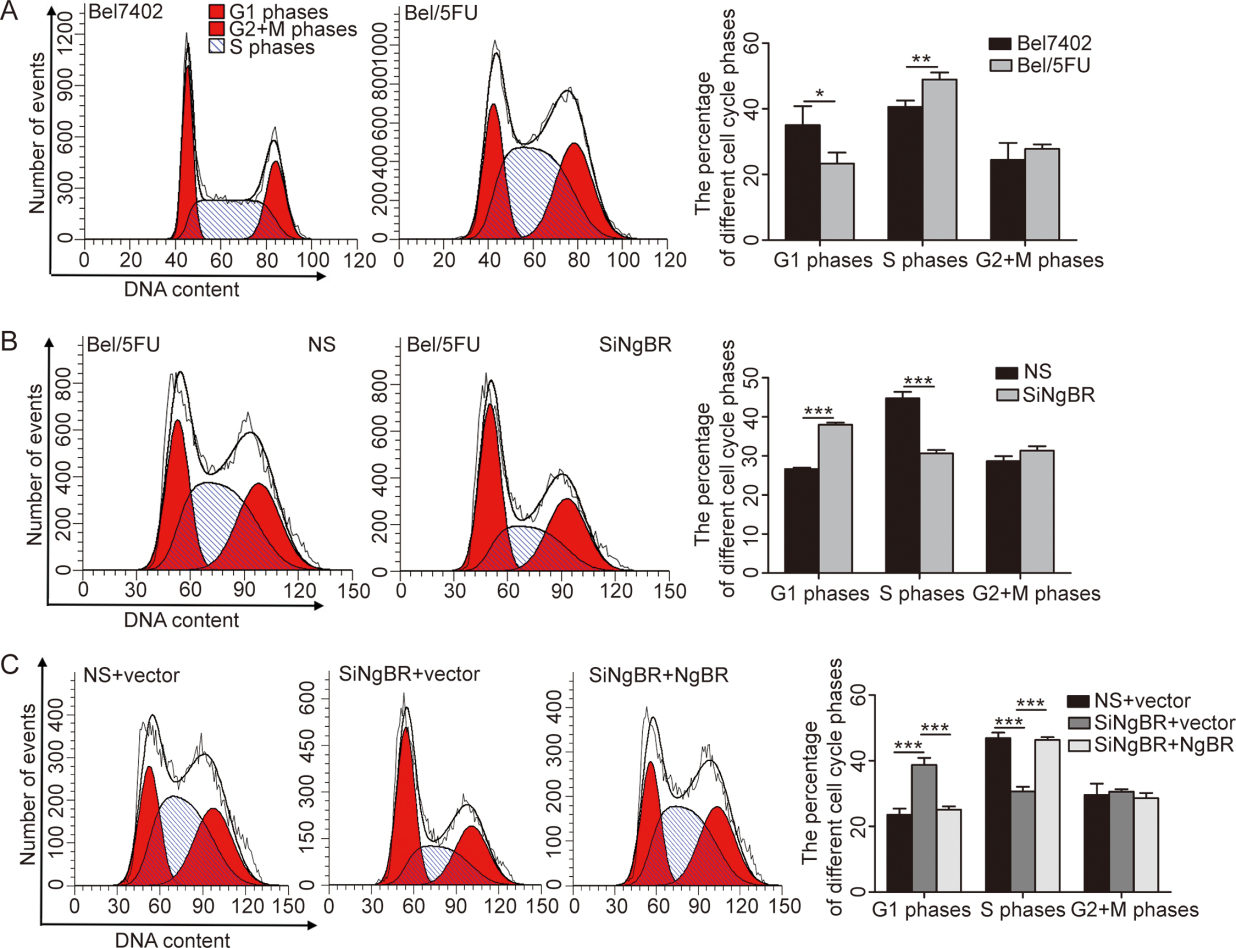
Knockdown of NgBR abrogates the S phase arrest in Bel/5FU cells (**A**) Bel/5FU cells undergo S phase arrest. The cell cycle distribution in Bel7402 cells and Bel/5FU cells was analyzed by flow cytometry by using PI staining. Representative figure from three independent experiments is shown in left panel. The percentage of different cell cycle phases was drawn in histogram form to reflect the alteration of cell cycle phases (right panel). The mean and SD obtained from three independent experiments are plotted (*P < 0.05, **P < 0.01). (**B**) Knockdown of NgBR restores the S phase arrest in Bel/5FU cells. The cell cycle distribution in Bel/5FU cells was analyzed by flow cytometry by using PI staining. Results are shown as the mean ± SD of three independent experiments (***P < 0.001). (**C**) Overexpression of NgBR rescued the cell cycle distribution impaired by siNgBR in Bel/5FU cells. Bel/5FU cells were co-transfected with siNgBR or NS and NgBR expression vector pIRES-NgBR or pIRES empty vector. Cell cycle was measured using flow cytometry. The mean and SD obtained from three independent experiments are plotted (***P < 0.001).

Cell-cycle dysregulation is a hallmark of cancer cells. Cell-cycle checkpoint protein dysfunction can alter the chemoresistance of cancer cells to chemotherapeutics [21]. To explore whether the chemoresistance of Bel/5FU cells is caused by cell cycle change, we examined the percentages of cell cycle distribution by PI staining-based FACS analysis. The results (Figure 3A) show that more Bel/5FU cells undergo S-phase arrest compared with the Bel7402 cells. Interestingly, knockdown of NgBR abrogated the S phase arrest in Bel/5FU cells (Figure 3B). Furthermore, by restoring NgBR expression by transfection of pIRES-NgBR, an NgBR-expressing plasmid, we found that NgBR overexpression rescues the abrogation of S-phase arrest presented in Bel/5FU NgBR knockdown cells (Figure 3C). These results suggest that chemoresistance of Bel/5FU cells may be caused by S phase arrest, and knockdown of NgBR abrogates this S phase arrest thus decreasing the chemoresistance of the Bel/5FU cells.

**Figure 4:**
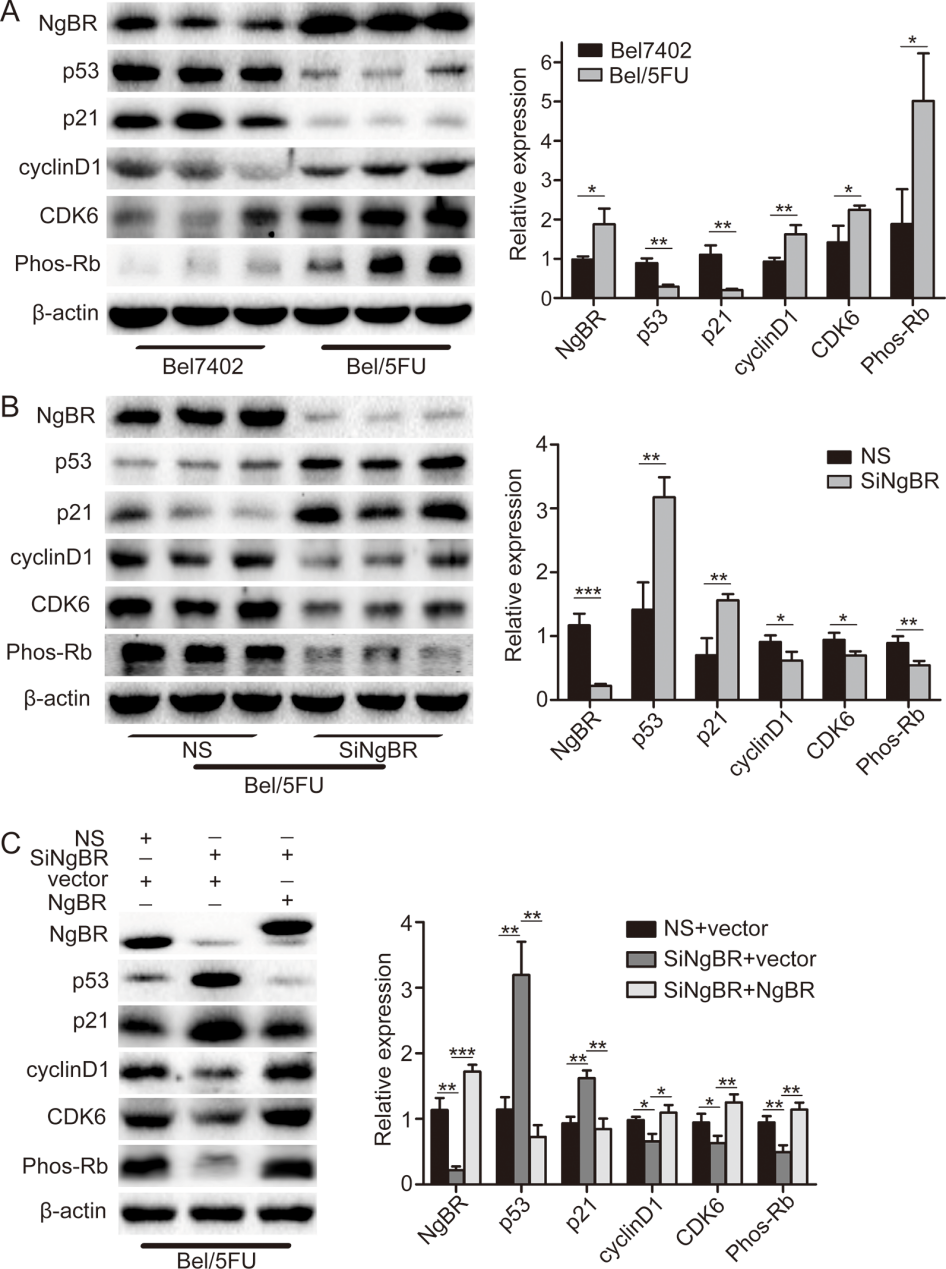
NgBR regulates p53 protein expression in Bel/5FU cells (**A**) p53 protein was decreased in Bel/5FU cells. p53, p21, cyclinD1, CDK6, and Phos-Rb protein levels in Bel7402 cells and Bel/5FU cells were determined using western blot analysis (left panel). Band intensities were quantified using Image Lab 5.0 software and were normalized to β-actin (right panel). The data are presented as the mean ± SD of three separate experiments. (*P < 0.05, **P < 0.01). (**B**) Knockdown of NgBR increases p53 protein level in Bel/5FU cells. The expression of p53, p21, cyclinD1, CDK6, and Phos-Rb in Bel/5FU cells were detected by western blot assay (left panel). The quantitative measurement is shown in right panel. The mean and SD obtained from three independent experiments are plotted (*P < 0.05, **P < 0.01, ***P < 0.001). (**C**) Overexpression of NgBR rescued the p53 protein level change induced by NgBR siRNA in Bel/5FU cells. The Bel/5FU cells were co-transfected with siNgBR or NS as well as pIRES-NgBR or pIRES empty vector. Expression of p53, p21, cyclinD1, CDK6, and Phos-Rb proteins was then examined using western blot analysis (left panel). The quantitative measurement is shown in right panel. The mean and SD obtained from three independent experiments are plotted (*P < 0.05, **P < 0.01, ***P < 0.001).

Given the S phase arrest, p53 protein and downstream p21 protein are the most important G1/S phase checkpoint proteins involved [22]. p21 binds to the CDK6/CyclinD1 complex and blocks the kinase activity of CDKs [23]. The CDK6/CyclinD1 complex increases phosphorylated Rb protein level followed by cell entry into S phase from G1 phase [21]. Therefore, we examined the p53 and p21 protein level in Bel7402 and Bel/5FU cells by western blot. As shown in Figure 4A, the p53 protein and downstream protein p21 protein level were decreased in Bel/5FU cells as compared to the control Bel7402 cells. Consequently, CDK6 and CyclinD1 protein expression as well as phosphorylated Rb protein levels are increased in Bel/5FU cells as compared to the control Bel7402 cells (Figure 4A). On the contrary, knockdown of NgBR in Bel/5FU cells increased p53 and p21 protein levels and consequently decreased CDK6, CyclinD1, and phosphorylated Rb protein levels as compared to the control Bel/5FU NS cells (Figure 4B). Furthermore, these protein level changes in NgBR knockdown Bel/5FU cells can be rescued by co-transfecting pIRES-NgBR plasmid DNA and siNgBR together as shown in Figure 4C. Collectively, these results suggest the altered cell cycle caused by decreased expression of p53 proteins and consequent reduction of p53 downstream signal pathway contributes to the chemoresistance of Bel/5FU cells. Knockdown of NgBR in Bel/5FU cells restores the p53 protein expression as well as normal cell cycle function and thus decreases the chemoresistance of Bel/5FU cells.

### NgBR promotes the ubiquitination of p53 via Akt-mediated MDM2 phosphorylation

**Figure 5:**
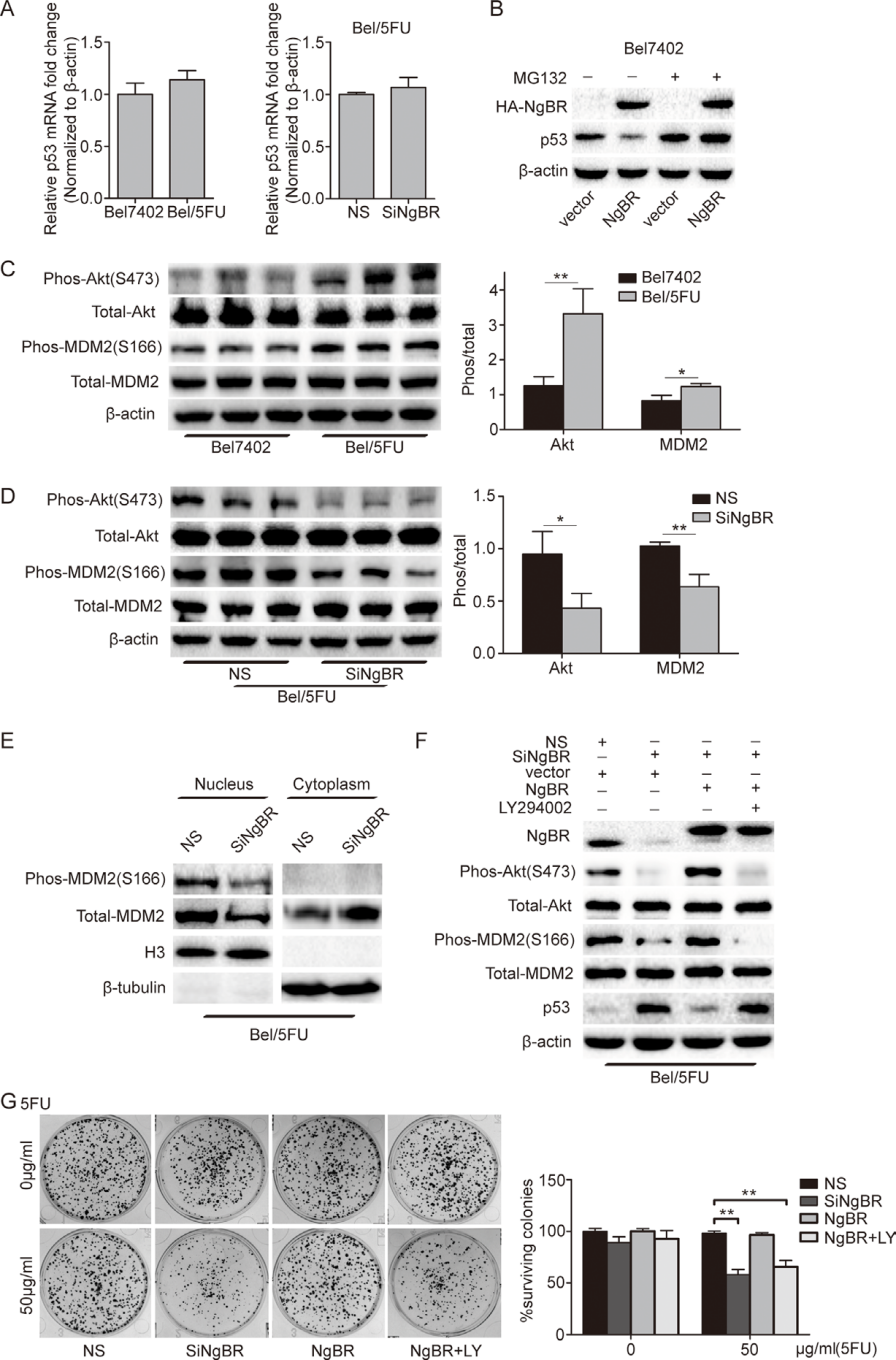
NgBR inhibits p53 expression by activating the PI3K/Akt/MDM2 mediated ubiquitin proteasome pathway (**A**) The mRNA level of p53 is not changed in Bel/5FU cells. The mRNA level of p53 in Bel7402 cells and Bel/5FU cells was analyzed using real-time RT-PCR (left panel). Bel/5FU cells were transfected with the indicated siRNA and then the mRNA level of p53 was analyzed by real-time RT-PCR (right panel). The relative amount of p53 mRNA level was normalized to the β-actin. (**B**) NgBR negatively regulates p53 expression in a proteasome dependent manner. Bel7402 cells were transfected with pIRES-NgBR or pIRES empty vector plasmid DNA for 48 h and then incubated with or without MG132 (20 μM) for an additional 4 h. Whole-cell lysates were analyzed by western blotting. (**C**) Phos-Akt and phos-MDM2 levels are increased in Bel/5FU cells. The phos-Akt and phos-MDM2 levels were assessed by western blotting. Total Akt and total MDM2 protein levels were used as a loading control (left panel). Band intensities were quantified using Image Lab 5.0 software(right panel). Results are shown as the mean ± SD of three independent experiments (*P < 0.05, **P < 0.01). (**D**) Knockdown of NgBR decreases the phos-Akt and phos-MDM2 level in Bel/5FU cells. Phos-Akt and phos-MDM2 levels were detected by western blot assay (left panel). The expression of each protein was quantified as the densitometry value analyzed by Image Lab 5.0 software and is normalized to total Akt and total MDM2 (right panel). The data are presented as the mean ± SD of three separate experiments. (*P < 0.05, **P < 0.01). (**E**) NgBR knockdown decreases the translocation of MDM2 from the cytoplasm to the nucleus. Localization of total MDM2 and phos-MDM2 proteins in nuclear (N) and cytoplasmic (C) cell fractions from Bel/5FU NS and Bel/5FU siNgBR cells was examined by western blot assay. Histone H3 and β-tublin were used as internal markers for the nucleus and cytoplasm, respectively. (**F**) NgBR regulates phos-MDM2 via Akt pathway. The Bel/5FU cells were co-transfected with siNgBR or NS with pIRES–NgBR plasmid or pIRES empty vector for 24 h and then incubated with or without LY294002 (25 μM) for an additional 48 h. The levels of phos-Akt, phos-MDM2, and p53 were detected by western blot assay. Total Akt, total MDM2, and β-actin protein levels were used as loading control. (**G**) NgBR regulates chemoresistance of Bel/5FU cells via Akt pathway. Clonogenic formation assay was used for measuring clonogenicity of Bel/5FU cells. The number of untreated cells is set as 100% and the results show the average percentage of surviving colonies. The data are presented as the mean ± SD of three independent experiments. (**P < 0.01, ***P < 0.001).

To further assess the mechanisms by which NgBR reduces p53 protein level, we investigated whether the p53 mRNA levels were changed in Bel/5FU cells first. The results showed that the p53 mRNA levels were not changed in Bel/5FU cells as compared with Bel7402 cells. Nor does knockdown of NgBR in Bel/5FU cells change the p53 mRNA level (Figure 5A). These results indicate that NgBR does not affect p53 transcript levels.

Previous reports [24, 25] showed that p53 protein undergoes degradation via the ubiquitin proteasome pathway. To determine the role of NgBR in regulating ubiquitination and degradation of p53 proteins in HCC cells, the degradation dynamics assay was used to detect the p53 protein level in Bel7402 and Bel/5FU cells. Cycloheximide(CHX) inhibits new protein translation. Our results show that the p53 protein diminished in control Bel/5FU NS cells 30 minutes after CHX treatment. However, the half-life of the p53 protein was increased in the NgBR knockdown Bel/5FU cells, and p53 protein expression was still detected after 2 hours of CHX treatment (Supplementary Figure S3A). In addition, we found that p53 protein was decreased in Bel7402 cells with pIRES-NgBR plasmid overexpression of NgBR (Figure 5B). However, if we treated the cells with the proteasome inhibitor MG132, NgBR overexpression does not change the p53 protein level as compared to Bel7402 cells transfected with control empty vector (Figure 5B). Furthermore, ubiquitin antibody immunoprecipitation results show that NgBR knockdown reduces p53 ubiquitination in Bel/5FU cells (Supplementary Figure S3B). These results suggest that NgBR regulates p53 protein level by promoting the degradation of p53 via ubiquitin proteasome pathway.

MDM2, an ubiquitin ligase for p53, plays a central role in the regulation of p53 protein stability. Previous studies have demonstrated Akt mediated phosphorylation of MDM2, allowing ubiquitination and degradation of p53 [26–29]. And It has been demonstrated that phosphorylation of Akt increased the translocation of MDM2 from the cytoplasm to the nucleus [30]. Our previous work has reported that NgBR regulates the Akt phosphorylation [18, 20]. Therefore, we hypothesized that NgBR regulates p53 protein stability via the PI3K/Akt/MDM2 signaling pathway in HCC cells. To test this hypothesis, we first used western blot analysis to detect the phosphorylation levels of both Akt and MDM2 in Bel7402 and Bel/5FU cells. The results showed that the phos-Akt and phos-MDM2 levels in Bel/5FU cells were higher than in the control Bel7402 cells, while the total Akt and MDM2 remained unchanged (Figure 5C). Knockdown of NgBR in Bel/5FU cells by siRNA significantly decreased the phosphorylation of both Akt and MDM2 levels while the total Akt and MDM2 remained unchanged (Figure 5D). In addition, NgBR knockdown decreases the translocation of MDM2 from the cytoplasm to the nucleus in Bel/5FU cells (Figure 5E). Furthermore, overexpression of NgBR by co-transfected pIRES-NgBR plasmid together with siNgBR in Bel/5FU cells can rescue impaired phosphorylation of both Akt and MDM2 levels in NgBR knockdown Bel/5FU cells (Figure 5F). While treatment with the PI3K inhibitor, LY294002, inhibits the levels of p-Akt and p-MDM2 in Bel/5FU cells highly expressing NgBR (Figure 5F). Meanwhile, a clonogenic survival assay showed that LY294002 also inhibits the rescue effects of NgBR overexpression in NgBR knockdown Bel/5FU cells (Figure 5G). Collectively, our results demonstrate that NgBR inhibits p53 expression by activating the PI3K/Akt/MDM2 pathway to promote the degradation of p53 via ubiquitin proteasome pathway and thus increases the chemoresistance of Bel/5FU cells.

### Knockdown of NgBR decreases tumor chemoresistance to 5-FU by increasing p53 protein leves in hepatocellular carcinoma *in vivo*

**Figure 6:**
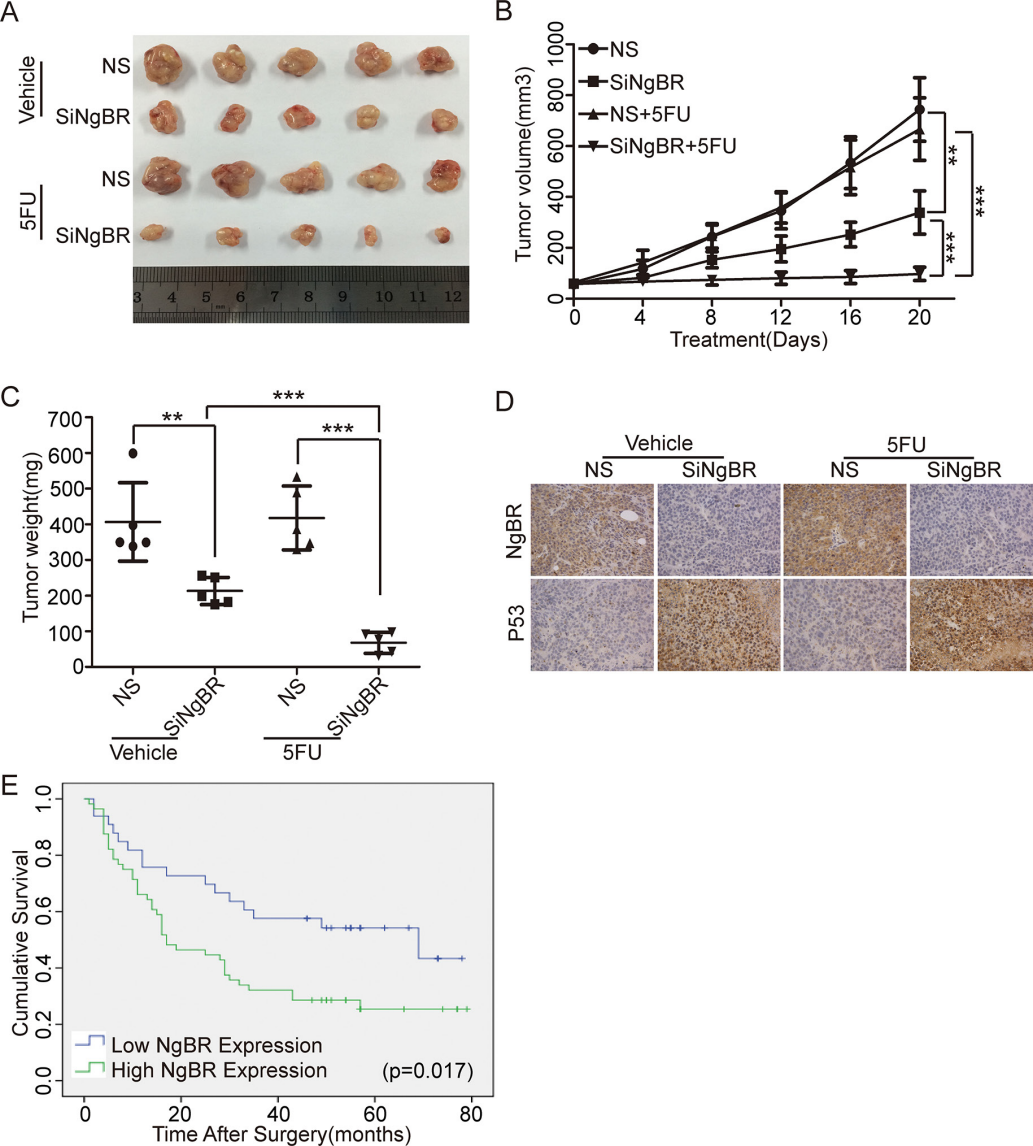
NgBR deficiency reverses the chemoresistance of human hepatocellular drug-resistant tumor to 5-FU through regulating p53 expression *in vivo* (**A**) Knockdown of NgBR decreases the tumor size. The representative images of Bel/5FU xenograft tumors injected intratumorally with non-silencing control siRNA (NS) or NgBR siRNA (siNgBR) and treated with vehicle or 5-FU for 3 weeks. (**B**) Knockdown of NgBR decreases the tumor volumes. Tumor volumes of different tumor and treatment groups were calculated as described in methods. The data are presented as the mean ± SD of three independent experiments. (**P < 0.01, ***P < 0.001). (**C**) Knockdown of NgBR decreases the tumor weights. The data are presented as the mean ± SD of three independent experiments. (**P < 0.01, ***P < 0.001). (**D**) Knockdown of NgBR increases p53 protein level *in vivo*. Representative immunohistochemical staining images showing the staining of NgBR and p53 in tumors tissue samples obtained from treatment groups at the end point. Scale bars, 50 μm. (**E**) NgBR expression is negatively associated with overall survival of human HCC patients. Kaplan-Meier analysis of overall survival with high or low NgBR expression of 89 primary human HCC patients (*P* = 0.017, log-rank test).

To further examine the *in vivo* role of NgBR in HCC chemoresistance to 5-FU treatment, we formed tumor xenografts by injecting Bel/5FU cells into nude mice. As shown in Figure 6A–6C, the growth of tumor xenografts was slower in the NgBR siRNA injected group, and 5-FU treatment further decreased the tumor size and weight dramatically in NgBR siRNA injected tumor xenografts. However, 5-FU treatment alone did not decrease tumor size or weight in the NS siRNA injected tumor xenografts, which confirms the chemoresistance of the Bel/5FU tumor xenografts *in vivo*. Further analysis of the tumor samples by IHC (Figure 6D) confirmed that NgBR reduction by injection of siNgBR significantly stabilized p53 expression in tumor xenografts. Collectively, our data strongly suggest that NgBR knockdown reduces the chemoresistance of human hepatocellular drug-resistant tumors to 5-FU through stabilizing p53 protein levels *in vivo*.

### NgBR is highly expressed in human HCC patient tissues and associated with a poor prognosis of HCC patients

To explore whether NgBR is an important factor in determining clinical outcomes of HCC patients, we examined the expression of NgBR in 89 primary HCC patient tissues and their corresponding adjacent liver tissues in a tissue microarray by IHC. Positive immunoreactivity for NgBR was observed primarily in the cell membrane and cytoplasm (Supplementary Figure S4A). As shown in Table 1, NgBR was highly expressed in HCC tissues compared with their corresponding adjacent liver tissues (*P* = 0.002). Segregation of patients into NgBR-positive and NgBR-negative groups did not reveal NgBR expression significant correlations with clinical pathological parameters of sex, gender, hepatitis history, liver cirrhosis, maximal tumor size, tumor number, vascular invasion, or TNM stage (Table 2). Furthermore, the overall survival analysis indicated patients with low NgBR expression owned significantly higher survival rates compared to the patients with high NgBR expression (*P* = 0.017) (Figure 6E). In addition, multivariate analyses revealed that vascular invasion and TNM stage, which are the established prognostic predictors for HCC [31], are independent prognostic factors for patient survival (Table 3). Our results indicate that NgBR is an independent prognostic factor for overall survival (hazard ratio, 2.255; 95% confidence interval, 1.209–4.206; *P* = 0.011) (Table 3). These results demonstrate that high NgBR expression is important in tumor progression and serves as an independent molecular marker for poor HCC prognosis.

**Table 1:** NgBR expression in tumor and adjacent tissues of liver cancer

		Adjacent tissues		*n*	*P*
		Low	High		
**Tumor tissues**	**Low**	21	12	33	0.002*
	**High**	16	40	56	
*n*		37	52	89	

**Table 2:** Correlation between NgBR expression determined by immunohistochemical staining and clinicopathological parameters in patients with liver cancer

Clinicopathological parameters		*n*	NgBR expression		*P*
			Low	High	
**Age (y)**					
	≤ 54	47	22	25	0.051
	> 54	42	11	31	
**Gender**					
	Male	80	29	51	0.906
	Female	9	4	5	
**Hepatitis history**					
	No	27	8	19	0.474
	Yes	62	25	37	
**Cirrhosis**					
	No	27	8	19	0.474
	Yes	62	25	37	
**Tumor size**					
	≤ 5 cm	38	13	25	0.306
	> 5 cm	51	20	31	
**Tumor number**					
	Single	46	15	31	0.389
	Multiple	43	18	25	
**Vascular invation**					
	No	78	30	48	0.700
	Yes	11	3	8	
**TNM stage**					
	I + II	46	19	27	0.511
	III + IV	43	14	29	

**Table 3:** Multiple analysis of factors associated with overall survival

Variable	Hazard Ratio	95% CI	*P*
**NgBR expression**	2.255	1.209 to 4.206	0.011*
**Age**	0.993	0.967 to 1.019	0.580
**Gender**	1.184	0.455 to 3.079	0.730
**Hepatitis history**	2.025	0.944 to 4.345	0.070
**Cirrhosis**	2.025	0.944 to 4.345	0.070
**Tumor size**	1.010	0.944 to 1.080	0.775
**Tumor number**	0.719	0.359 to 1.437	0.350
**Vascular invation**	3.117	1.385 to 7.018	0.006*
**TNM stage**	2.145	1.323 to 3.479	0.002*

## DISCUSSION

The development of drug resistance is the major obstacle in successful and effective chemotherapeutic treatment of HCC [32]. HCC frequently and easily acquires chemoresistance. Therefore, conventional chemotherapy treatments achieve poor efficacy in patients with advanced HCC and often do not show any benefit to survival [33]. The mechanism by which HCC acquires chemoresistance is still not well understood. In this study, we demonstrate that NgBR expression is upregulated in HCC cells with acquired chemoresistance and knockdown of NgBR in the chemoresistant Bel/5FU cells reduces the chemoresistance in HCC cells. These results indicate that NgBR could play an important role in the development of chemoresistance in HCC cells.

5-Fluorouracil (5-FU) is widely used in the treatment of a variety of tumors. It interferes with nucleoside metabolism and results in DNA synthesis disorders and RNA dysfunction, leading to cytotoxicity [34]. However, chemotherapeutic drugs that function by inducing DNA damage frequently fail due to tumors acquiring the ability to repair the DNA damage and therefore escaping cell death. Cell cycle dysregulation has been demonstrated to be involved in the growth inhibition and DNA repair by DNA-damaging drugs [35]. Alterations in expression of proteins that control progression through the cell cycle have been demonstrated to affect chemosensitivity [36]. Mutated forms of p53 and/or Rb have been shown to be associated with increased resistance of tumor cells to various anticancer drugs and irradiation, mainly because of cell cycle dysregulation [37]. Furthermore, alterations in other cell cycle regulators such as the cyclins, cyclin-dependent kinases (CDKs), and their inhibitors, p21^Cip1/WAF1^ and p27^Kip1^, may also play an important role in the regulation of drug sensitivity [38]. In this study, we show that chemoresistant Bel/5FU cells undergo an S-phase arrest not seen in Bel7402 parental cells. Surprisingly, we found that knockdown of NgBR abrogates the S-phase arrest in Bel/5FU cells. In addition, we also clearly demonstrate that altered expression of these cell cycle regulators is triggered by NgBR, which is consistent with alterations of the cell cycle distribution. Therefore, we conclude that targeting NgBR could partially reverse the drug resistance of Bel/5FU cells by regulating cell cycle regulators, which results in alterations of the cell cycle progression.

p53 suppresses cancer progression through the induction of cell cycle arrest, apoptosis, or senescence in response to a variety of cellular stimuli. As we know, p53 acts as the most important checkpoint protein to suppress cell growth by inhibiting G1 progression to S phase while the cell attempts to repair the damage or to promote apoptosis in cells that fail to repair [39, 40]. Cancer cells harboring wild-type p53 are generally sensitive to antitumor agents [41]. In contrast, loss of p53 function in cells, either through mutation or post-translational modification, might therefore be expected to lead to unchecked proliferation, tumor growth, and therapeutic resistance [42, 43]. In addition, a report showed that p53 gene transfection not only induced suppression of cell growth, but also increased the sensitivity of Bel/5FU cells to 5-FU, vincristine, and doxorubicin [32]. In this study, we found a robust loss of p53 in chemoresistant HCC cells (Bel/5FU) compared with their corresponding parental cells (Bel7402). Interestingly, we found that p53 protein levels increased upon knock down of NgBR in Bel/5FU cells.

**Figure 7:**
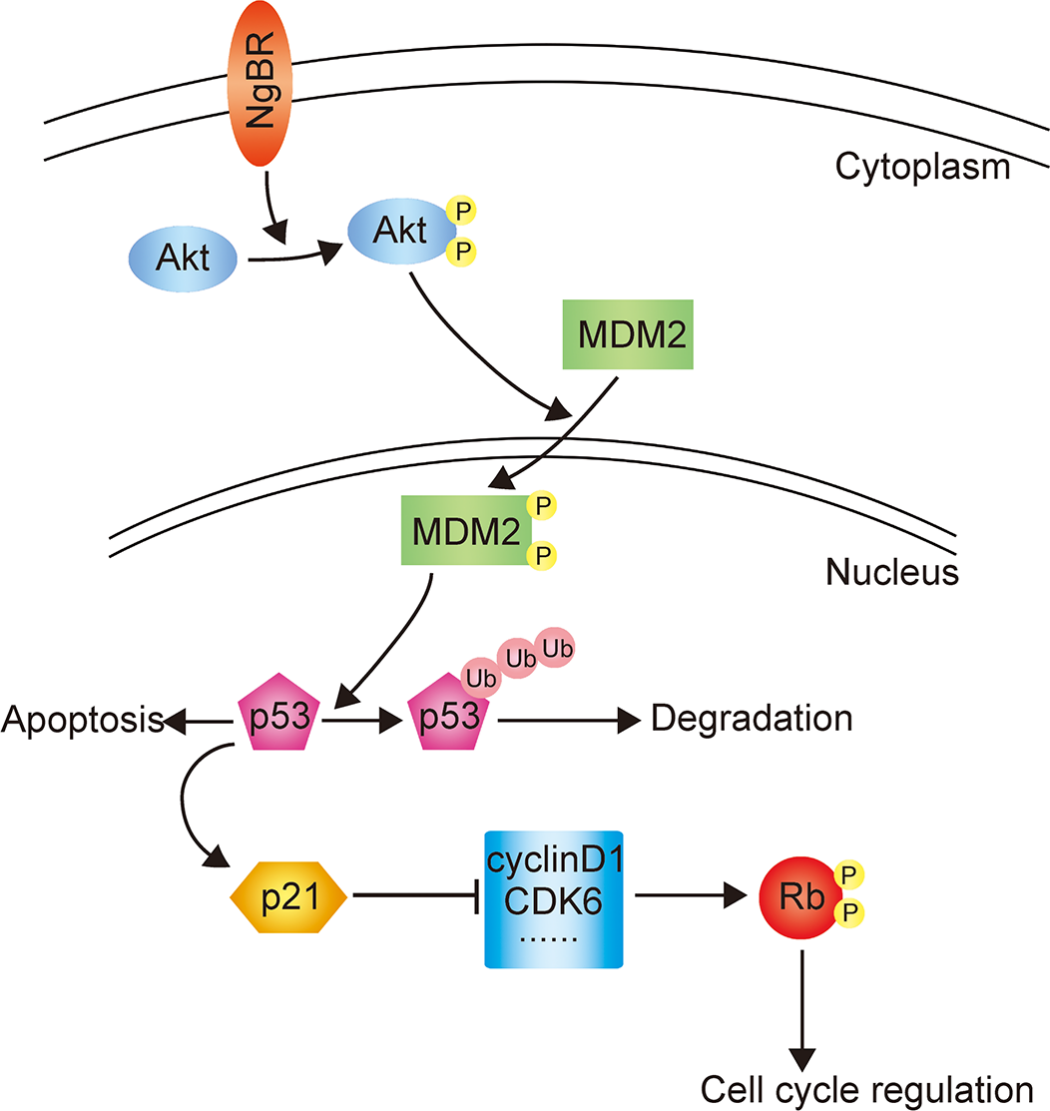
The proposed working model elucidating the roles of NgBR in regulating the chemoresistance of HCC cells

Previous studies have demonstrated that Akt mediates phosphorylation of MDM2 and induces its nuclear translocation, allowing ubiquitination and degradation of p53 [26–29]. Our previous work has reported that NgBR regulates the Akt phosphorylation [18, 20]. In this study, we found that NgBR increases the level of phos-Akt; phos-Akt then phosphorylates MDM2, and phos-MDM2 facilitates the ubiquitination and degradation of p53 proteins. In conclusion, we show that NgBR facilitates ubiquitination and degradation of p53 proteins via the PI3K/Akt/MDM2 mediated ubiquitin proteasome pathway and thus increases the drug resistance of Bel/5FU cells. The proposed working model showing the roles of NgBR in regulating the chemoresistance of HCC cells is shown in Figure 7.

In summary, our results demonstrate that higher NgBR expression in HCC cells contributes to 5-FU chemoresistance both *in vitro* and *in vivo*. This study provides compelling evidence in support of targeting NgBR in combination with 5-FU as a viable option for improved treatment of drug resistant HCC.

## MATERIALS AND METHODS

### Chemicals and reagents

5-Fluorouracil (5-FU) was purchased from Sigma Aldrich (St. Louis, MO, USA). The Cell Counting Kit-8 and Annexin V-FITC/PI Apoptosis Detection Kit were purchased from Dojindo (Kumamoto, Japan). Co-immunoprecipitation Kit was purchased from Thermo (Rockford, IL, USA). LY294002 and MG132 were purchased from Selleck (Houston, TX, USA). All the other chemicals and reagents were purchased from Sigma (St. Louis, MO, USA) unless otherwise specified. All the water used in experiments was purified using a Milli-Q system (Millipore, Bedford, MA, USA).

### Antibodies

NgBR rabbit monoclonal antibody (CloneID: EPR8668) was generated by Epitomics (Burlingame, CA, USA) as a collaboration project. NgBR rabbit polyclonal antibody was generated as described previously [19] and was used for immunohistochemistry analysis. Rabbit polyclonal antibodies for phospho-AKT (Ser473), total Akt, Phospho-MDM2 (Ser166), cyclinD1, p21, phos-Rb (Ser807/811), HA-Tag rabbit monoclonal antibody, Histone H3, and β-actin and all the secondary antibodies were purchased from Cell Signaling (Danvers, MA, USA). Rabbit polyclonal antibodies for p53, ubiquitin, CDK6, and β-tublin were purchased from ProteinTech (Chicago, IL, USA). Rabbit polyclonal antibody for total MDM2 was purchased from Anbo Biotechnology (San Francisco, CA, USA).

### Cell lines and cell culture

Human HCC cell lines Bel7402 and 5-FU-selected drug-resistant Bel7402/5FU (Bel/5FU) were obtained from KeyGen Biotech Co., Ltd (Nanjing, China). The cell lines were cultured in RPMI Medium 1640 (Gibco, USA) supplemented with antibiotics(1 × penicillin/streptomycin 100 U/ml, Gibco, USA) and 10% fetal bovine serum (Gibco, USA). To maintain the drug-resistant phenotype, the medium for the Bel/5FU cells was supplemented with 20 μg/ml 5-FU. Cells were incubated at 37°C in a humidified atmosphere containing 5% CO2.

### Transfection of siRNA and plasmid DNA

NgBR siRNA1 (S1forward: GGAAAUACAUAGA CCUACA, S1reverse: UGUAGGUCUAUGUAUUUCC), NgBR siRNA2 (S2 forward: GUAUGGAAAUAAACU UAUA, S2 reverse: UAUAAGUUUAUUUCCAUAC) and NgBR siRNA3 (S3 forward: GCUGAUUCUUAGAU AGAAA, S3 reverse: UUUCUAUCUAAGAAUCAGC) oligonucleotides with 3′dTdT overhangs were synthesized by Shanghai GenePharma Co. (Shanghai, China). Control siRNA in experiments refers to an All-Star non-silencing siRNA (forward sequence:GGGUAUCGACGAUUACA AAUU, reverse sequence: UUUGUAAUCGUCGAUA CCCUG) synthesized by Shanghai GenePharma Co. (Shanghai, China). Lipofectamine RNAiMAX reagent (Invitrogen, Carlsbad, CA, USA) was used for the transfection of siRNA according to the manufacturer's instructions. Lipofectamine 2000 (Invitrogen, Carlsbad, CA, USA) was used for transfection of NgBR expression plasmid pIRES-NgBR. The specificity of NgBR siRNA and pIRES-NgBR has been validated in our previous publications [17, 18].

### Quantitative real-time polymerase chain reaction

Total RNA was extracted from cells by using TRIzol reagent according to the manual (TaKaRa Bio, Dalian, China) and cDNA was reverse-transcribed using the PrimeScript RT Reagent Kit (TaKaRa Bio, Dalian, China) according to the manufacturer's instructions. Real-time PCR was performed using QuantiTect SYBR Green PCR kit (TaKaRa Bio, Dalian, China) and was run on Stratagene MX3000P (Agilent, CA, USA). The relative mRNA expression of each gene was normalized to β-actin RNA levels. The primers were synthesized by Invitrogen (Carlsbad, CA, USA). The forward and reverse primers for NgBR are 5′-tgccagttagtagcccagaagcaa-3′ and 5′-tgatgtgccagggaagaaagccta-3′, respectively. The forward and reverse primers for p53 are 5′-gcgtgtggagtatttggatgac-3′ and 5′-agtgtgatgatggtgaggatgg-3′, respectively. The forward and reverse primers for β-actin are 5′-ttctacaat gagctgcgtgtggct-3′ and 5′-tagcacagcctggatagcaacgta-3′ respectively.

### Western blot

Cells were harvested and lysed in immune precipitation assay buffer (KeyGen Biotech Co., Ltd, Nanjing, China) supplemented with 1 mM phenylmethylsulfonyl floride (KeyGen Biotech Co., Ltd, Nanjing, China) and 1 mM phosphatase inhibitor cocktail (KeyGen Biotech Co., Ltd) for extracting whole cell protein samples. For detecting the cellular localization of total MDM2 and phos-MDM2, nuclear and cytoplasmic fractions were isolated using the Nuclear and Cytoplasmic Protein Extraction kit (Thermo Fisher Scientific) according to the instructions of the manufacturer. Protein concentration was determined using a BCA protein assay kit (KeyGen Biotech Co., Ltd). Same amount protein samples were separated on 10% SDS-PAGE gels and then transferred to a polyvinylidene difluoride membrane (Pall Corporation, Port Washington, NY, USA). The protein band intensities were evaluated using ECL western blotting kit (Advansta, Menlo Park, CA, USA) and were normalized to those of β-actin. All experiments were performed at least three times.

### Clonogenic survival assay

Cells were seeded in triplicate into a 6-well culture dish (1000 cells/well). Cells were transfected with NgBR siRNA and/or NgBR expressing vector pIRES-NgBR. At 24 hours after transfection, cells were treated with 5-FU and/or Akt inhibitor LY294002 at the indicated doses for 72 hours. Then, the cells were maintained for 2 weeks. The cell colonies were washed three times with phosphate buffered saline buffer (PBS), fixed in methanol for 15 minutes, and stained with Crystal Violet (Sigma-Aldrich, St. Louis, MO, USA) for 15 minutes at room temperature.

### Apoptosis assay by AO/EB staining

The cells cultured in 6-well plates were treated with different concentrations of 5-FU for 48 hours. After indicated treatment times, the cells were stained with acridine orange (AO, 200 μg/mL) and ethidium bromide (EB, 200 μg/mL) for 2 min and then washed with PBS to remove background staining. After that, cells were observed under a fluorescence microscope (Nikon Ti-S, Nikon Inc, Japan). The normal cells and early apoptotic cells can be stained by AO to display bright green fluorescence, while the late apoptotic cells can be stained by EB to display orange fluorescence.

### Apoptosis measured by annexin V-FITC/propidium iodide (PI) staining

An Annexin V-FITC/PI apoptosis kit was used to quantify the percentage of cells undergoing apoptosis. Cells cultured in 6-well plates were treated with 5-FU (50 μg/mL) for 48 hours. Cells were then harvested and stained with 5 μL Annexin V-FITC and 5 μL PI in 500 uL of apoptosis reaction solution at room temperature in the dark for 30 min. Accuri C6 flow cytometer (Accuri Cytometers, Inc., Ann Arbor, MI, USA) was used to detect apoptotic cells. Cell population in different quadrants was calculated statistically.

### Cell cycle analysis

The cells were treated with 5-FU at the indicated concentrations, harvested by trypsinization, and fixed overnight with 70% cold ethanol at −20°C. Fixed cells were stained with the 1 ml 50 μg/ml PI solution containing 0.1% Triton X-100 and 0.1 mg/ml RNase in the dark at 37°C for 30 min. The cell cycle profiles were obtained by Accuri C6 flow cytometer. ModFit LT software (Verify Software, Topsham, MN, USA) was used to calculate the percentages of cells in each cycle phase.

### Cell viability assay

Cell viability was determined using the CCK-8 assay according to the manufacturer's instructions. Cells were seeded in 96-well plates at 5000 cells/well. At 24 hours after seeding, cells were transfected with All Star NS siRNA or NgBR siRNA. Then, after the cells were treated with 5-FU (50 μg/ml) for 24, 48, or 72 hours, the medium was exchanged for 100 μl of RPMI-1640 and 10 μl of CCK-8 reagent was added. The cells were incubated for 3 hours at 37°C. The optical density was measured using an EnSpire^™^ 2300 Multilabel Reader (Perkin Elmer, Waltham, MA, USA) at 450 nm. Three replicates were prepared for each condition.

### Ubiquitination assays

Cells were transfected with indicated siRNA and cultured for 48 h, and then incubated with 20 μM MG132 for 4 h. Whole-cell lysates were harvested and used for immunoprecipitation (IP) to detect protein ubiquitination. The IP experiments were performed with the Pierce Co-IP Kit (Thermo Scientific) according to the manufacturer's protocol. 10 μg of the ubiquitin antibody were covalently coupled with the delivered resin. The antibody-coupled resin was incubated with 400 μg of the whole-cell protein lysates overnight at 4°C. The precipitated proteins were denatured and resolved by 2 × SDS-PAGE loading buffer. Ubiquitinated proteins were then detected by anti-p53 antibody.

### Tissue microarray and immunohistochemistry analysis

The HCC tissue microarray containing 89 primary HCC samples and their corresponding adjacent liver tissues was purchased from Shanghai Outdo Biotech (Shanghai, China). The overall survival (OS) for the corresponding patients was calculated from the day of surgery to the day of death or to the last follow-up. Immunohistochemistry was performed using a standard methodology to analyze NgBR protein expression. Briefly, the tissue microarray (TMA) slides were heated in citrate buffer (pH 6.0) in a pressure cooker for 6 min and incubated with the rabbit polyclonal anti-NgBR antibody (1:200) overnight at 4°C. The TMA were then incubated with goat anti-rabbit Envision System plus-HRP (Dako Cytomation) for 30 min at room temperature and counter stained with Mayer's hematoxylin. The degrees of immunostaining were reviewed and scored by two independent pathologists who were blinded to patient outcome. Cytoplasmic or membranous staining for NgBR was considered positive [19]. The proportion of the stained cells and the extent of the staining were used as criteria of evaluation. For each case, at least 1,000 tumor cells were analyzed and the percentage of the tumor cells with positively staining was recorded [44]. The percentage of positive cells was assigned a score from 0 (< 5%), 1 (5–25%), 2 (> 25–50%), 3 (> 50–75%), or 4 (> 75%) and the staining intensities within the respective subcellular locations were noted as 0 = negative, 1 = weak, 2 = moderate, or 3 = strong. A final score was then calculated by multiplying the above two scores. If the final score was equal or bigger than six, the protein expression in the tumor was considered high; otherwise, the protein expression in the tumor was considered low [19].

### Animal studies

The male nu/nu mice (4–6 weeks old) were used and all animal experiments were maintained in SPF Laboratory Animal Center at Dalian Medical University. Cholesterol-conjugated All Star non-silencing siRNA (NS) and NgBR siRNA (siNgBR) for *in vivo* delivery were obtained from Shanghai GenePharmaCo. (Shanghai, China). Bel/5FU cells (5 × 10^6^ in 100 μL PBS) were inoculated subcutaneously into the flank of the nude mice. Two weeks later, when the tumor diameters reached 4 mm to 5 mm, mice were randomly divided into 4 different groups *n* = 5/group): non-targeting siRNA injected group (NS), NgBR siRNA injected group (siNgBR), non-targeting siRNA injected plus 5-FU treatment group (NS + 5-FU) and NgBR siRNA injected plus 5-FU treatment group (siNgBR + 5-FU). 10 nmol indicated siRNA in 0.1 ml saline buffer was injected intratumorally twice a week for 3 weeks [44, 45]. At the same time, 30 mg/kg 5-FU in saline buffer was injected intraperitoneally three times per week for 3 weeks in the 5-FU treatment groups. Tumors were measured with a caliper every 4 days, and the tumor volume was calculated using the formula V = 1/2 (width^2^ × length). At 5 weeks after tumor cell inoculation, all mice were terminated with ether anesthesia and the total weight of the tumors in each mouse was measured. Tumor specimens were harvested for NgBR and p53 protein immunohistochemistry staining.

All animal maintenance and procedures were carried out in strict accordance with the recommendations established by the Animal Care and Ethics Committee of Dalian Medical University as well as the guidelines of the U.S. National Institutes of Health Guide for the Care and Use of Laboratory Animals. The protocol was approved by the Animal Care and Ethics Committee of Dalian Medical University.

### Statistical analysis

All experiments were repeated three times. The chi squared test, Analysis of Variance, and two-tailed Student *t* test were performed as appropriate. The cumulative survival probability was evaluated using the Kaplan-Meier method, and the differences were assessed using the log-rank test. Cox multivariate regression analysis was used to determine independent prognostic factors. A *p*-value < 0.05 defined statistical significance. SPSS 17.0 software was used for all statistical analysis.

## SUPPLEMENTARY MATERIALS FIGURES


