# Supplementary material for: Nogo-B receptor promotes the chemoresistance of human hepatocellular carcinoma via the ubiquitination of p53 protein
Source: Oncotarget. 2016 Jan 31;7(8):8850–65. doi: 10.18632/oncotarget.7091 (PMC4891009; doi:10.18632/oncotarget.7091)
Supplement: Supplementary file 1 [file oncotarget-07-8850-s001.pdf]

Nogo-B receptor promotes the chemoresistance of human hepatocellular carcinoma via the ubiquitination of p53 protein

Supplementary Materials

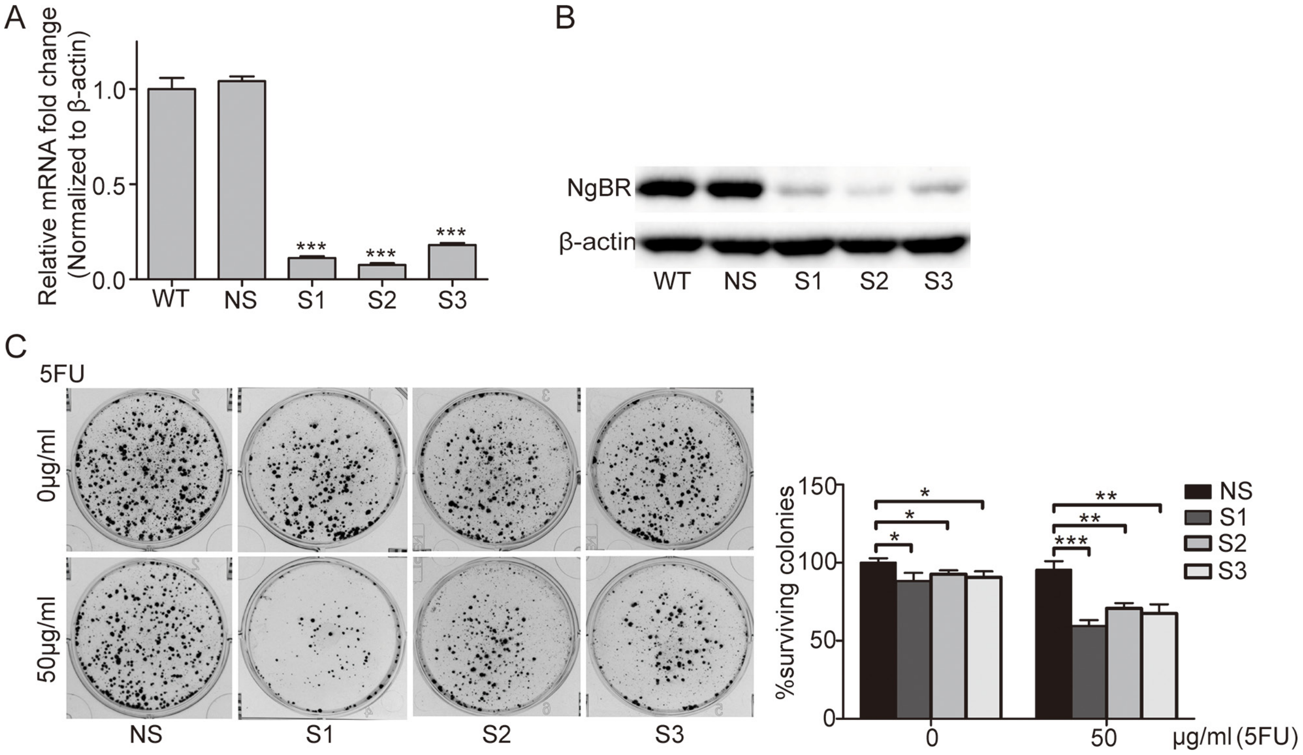

Supplementary Figure S1: Knockdown of NgBR decreases clonogenicity of Bel/5FU cells. (**A**) and (**B**) All three siRNA targeting NgBR knockdown NgBR effectively. The relative amount of NgBR mRNA level was normalized to the β-actin. (***$P < 0.001$) (A) NgBR protein level was determined using western blot analysis (B). (**C**) Knockdown of NgBR decreases clonogenicity of Bel/5FU cells. Clonogenic survival assay was used for measuring clonogenicity of Bel/5FU cells with 5-FU (0 and 50 μg/mL) treatment. The number of untreated cells is set as 100 % and the results show the average percentage of surviving colonies. The data are presented as the mean ± SD of three independent experiments. (*$P < 0.05$, **$P < 0.01$, ***$P < 0.001$).

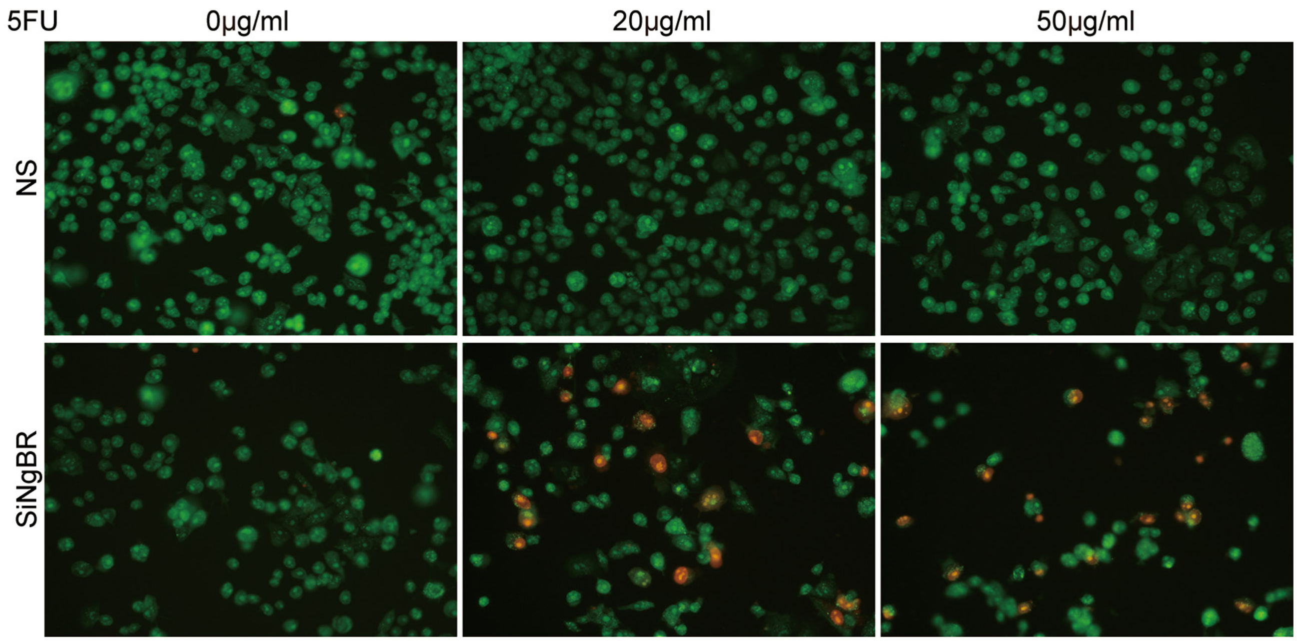

Supplementary Figure S2: NgBR knockdown enhances 5-FU-induced apoptosis of Bel/5FU cells. Representative images of AO/EB staining.

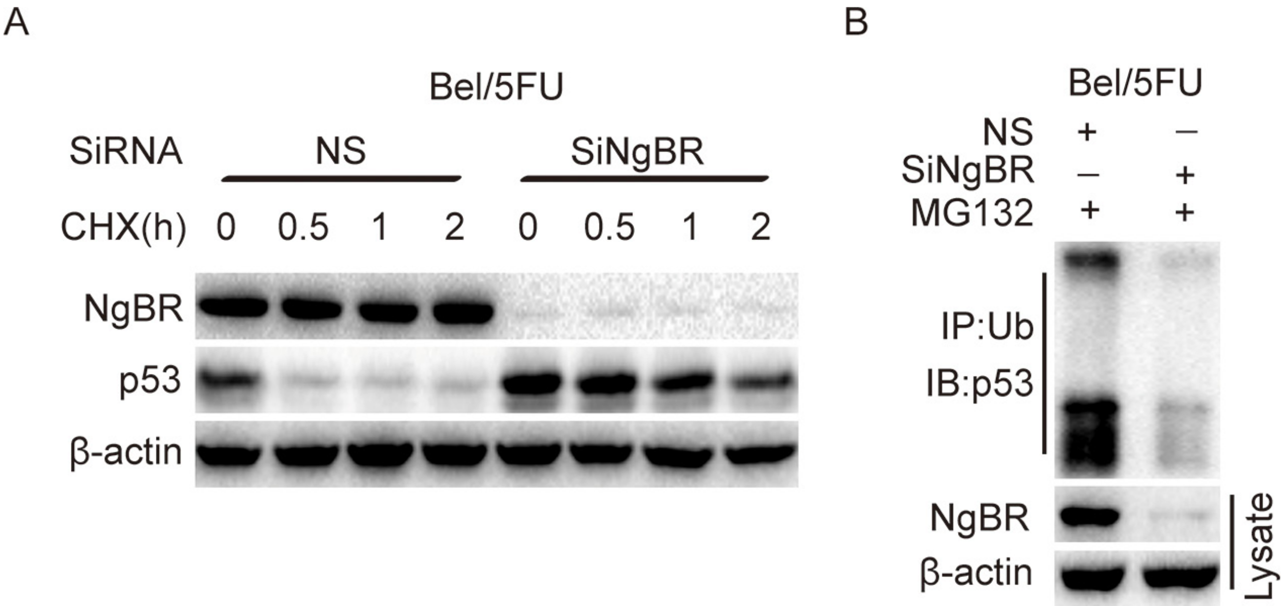

Supplementary Figure S3: Knockdown of NgBR inhibits the ubiquitination of p53 protein in Bel/5FU cells. (**A**) Knockdown of NgBR stabilizes p53 protein in Bel/5FU cells. Bel/5FU cells transfected with NS or siNgBR were treated with 20 μg/ml cycloheximide (CHX) and harvested at the indicated time points. Protein levels were analyzed by western blotting. (**B**) Knockdown of NgBR decreases the ubiquitination of endogenous p53. Bel/5FU cells transfected with NS or siNgBR and incubated with MG132 (20 μM) for an additional 4h. Whole-cell lysates were immunoprecipitated with an ubiquitin antibody and p53 protein were detected by western blotting.

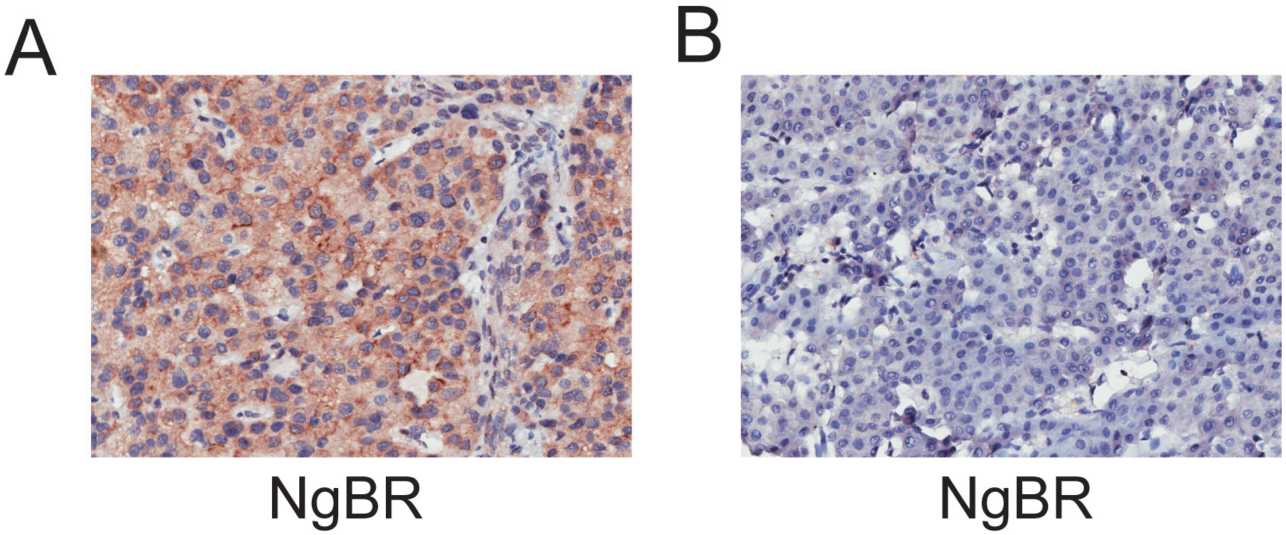

Supplementary Figure S4: IHC staining of NgBR in primary HCC tissue. Representative image of the positive (**A**) and negative (**B**) staining of NgBR in primary HCC tissue (magnification 200×).
